# Molecular Genetic Factors of Risk Stratification of Lymph Node Metastasis in Endometrial Carcinoma

**DOI:** 10.3390/cancers16213560

**Published:** 2024-10-22

**Authors:** Aida Gilyadova, Anton Ishchenko, Julietta Babayan, Max Avin, Marina Sekacheva, Igor Reshetov

**Affiliations:** 1 Federal State Autonomous Educational Institution of Higher Education First Moscow State Medical University Named after. I. M. Sechenov Ministry of Health of Russia (Sechenov University), Ministry of Health of the Russian Federation, 119435 Moscow, Russia; julietpochta@gmail.com (J.B.); maxavin82@gmail.com (M.A.); sekach_rab@mail.ru (M.S.); reshetoviv@mail.ru (I.R.); 2 National Medical Research Center Treatment and Rehabilitation Center, Ministry of Health of the Russian Federation, 125367 Moscow, Russia; ra2001_2001@mail.ru

**Keywords:** endometrial cancer, endometrial carcinoma, medical–genetic factors, DNA methylation, lymphadenectomy, microRNA expression, sentinel lymph node, tumor marker, genetic profiling

## Abstract

Background: According to epidemiological studies, endometrial carcinoma is one of the most frequently diagnosed malignancies of the female reproductive system, with an increasing incidence. Currently, the risk stratification of this neoplasm takes into account the stage, degree of tumor differentiation, histological type and depth of myometrial invasion. Since the publication of the last International Federation of Gynecology and Obstetrics (FIGO) staging system for endometrial cancer in 2009, numerous reports have appeared on the molecular characteristics of different types of endometrial carcinoma. Taking this into account, the FIGO Committee determined in 2023 that changes and updates to the staging system are justified to reflect new information about this tumor. Due to the high prevalence of the disease and mortality from endometrial cancer, an in-depth study of the molecular genetic characteristics of tumor cells is relevant; the results of such studies can be used to improve the efficiency of diagnosis, assess the risk of metastasis and prognosis of the disease. Lymph node assessment is crucial for the choice of treatment strategy for endometrial cancer, since metastatic lymph node involvement is one of the main factors affecting prognosis. At the same time, the criteria for the appropriateness of lymphadenectomy in low-differentiated malignant tumors are not clearly defined. Various molecular methods have been proposed to assess the status of lymph nodes; candidate genes are being studied as potential diagnostic biomarkers, as well as microRNA. The aim of the study was to analyze the literature data on numerous studies of molecular risk factors for progression in endometrioid carcinoma, as well as to preserve the most important marker changes in relation to the prognostic development of this disease. Methods: A literature review was conducted using data from the electronic databases PubMed, Google Scholar, and Wiley Cochrane Library for the period from 2018 to 2023 using the specific keywords. Results: The current scientific genetic studies on metastasis and prognostic factors in uterine cancer were analyzed, and a systematization of the reviewed data from the modern literature was done. Conclusions: To select the most effective treatment - intraoperative, adjuvant or combination therapy, minimize postoperative risks of lymphadenectomy and clearly predict the results - further study of the molecular genetic features of endometrial cancer is necessary.

## Simple Summary

Endometrial cancer is an important problem and is among the leading causes of reproductive system cancer in women. Every year, the number of cases of the disease only increases, and a high mortality rate from this disease is also recorded. The risk stratification of patients with endometrial carcinoma is based on the severity of the oncological process progression, which makes it necessary to study new diagnostic methods for the more accurate determination of lymph node involvement. Currently, new medical–genetic factors, which may be relevant for assessing the risk of metastasis and disease progression, are actively being studied. Candidate genes, microRNA, and other various biomarkers are being studied, promising the benefit of choosing more effective treatments.

## 1. Introduction

**Figure 1:**
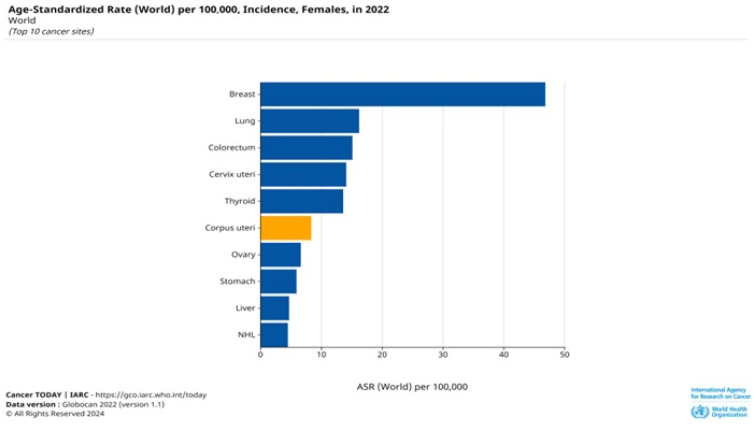
Incidence of corpus uteri cancer according to the International Agency for Research on Cancer 2022.

Endometrial cancer is one of the most common malignant tumors of the female reproductive system, accounting for 20–30% of gynecological cancers and 7% of all cancer cases in women [1]. It ranks sixth among the most diagnosed malignant tumors in women worldwide. According to the International Agency for Research on Cancer 2022 (Figure 1), the incidence of endometrial cancer amounts to 420,368 cases, which is 1.7 cases per 100,000 people, while the mortality from this pathology reached 97,370 cases in 2022 [2].

The classification of the types of endometrial cancer considering pathogenesis was proposed by Bokhman in 1983 [3]. This system suggested distinguishing between two types of endometrial cancer and has been widely used as a basis for refining prognosis and selecting appropriate treatment approaches that reflect the different pathogenesis within each of these groups. Endometrial cancer type I (70%) is primarily mediated by the consequences of obesity and is associated with the excessive proliferation of endometrial cells. Accordingly, patients with type I endometrial cancer often exhibit hyperestrogenism, hyperlipidemia, diabetes, and anovulatory uterine bleeding; all these conditions are associated with metabolic syndrome, which has been identified as an independent risk factor for the development of endometrial cancer [4]. Histologically, type I tumors are predominantly well- and moderately differentiated, and at least 90% of them express moderate to high levels of estrogen receptors [5]. Endometrial cancer type II, on the contrary, is not associated with hyperestrogenemia or endometrial hyperplasia, often occurring in women who are not obese, and is not linked to metabolic or endocrine disorders. Histologically, type II tumors are poorly differentiated, most often serous, clear cell, or subtypes of carcinosarcoma. They are clinically aggressive and associated with a more advanced stage at the initial consultation with a doctor and a higher risk of recurrence. Precancerous forms vary for each type: endometrial intraepithelial neoplasia is associated with type I cancer, while endometrial intraepithelial carcinoma is linked to type II [6]. Serous endometrial intraepithelial carcinoma (SEIC) is a noninvasive precursor of invasive serous carcinoma of the uterus. In SEIC, glandular epithelium is replaced by neoplastic cells without the invasion of the endometrial stroma, which is associated with endometrial atrophy or the development of an intra-endometrial polyp. Endometrioid intraepithelial neoplasia is a monoclonal noninvasive genetically altered neoplasia that focally develops from glandular cells and is manifested by impaired glandular proliferation and a shift in the glandular–stromal ratio in the endometrium, as well as the presence of nuclear atypia. Endometrial intraepithelial neoplasia indicates a high risk of developing endometrioid adenocarcinoma [7,8].

Endometrioid adenocarcinoma is the most common morphological form of endometrial cancer, occurring in 75–80% of cases [9]. Serous and clear cell cancers account for about 10% and 4%, respectively, are detected at an advanced stage, and have a worse prognosis at any stage compared to high or moderately differentiated endometrioid adenocarcinomas of a similar stage. At the time of presentation, 70% of serous carcinomas have already spread beyond the uterus, and extraperitoneal spread is often present even in the absence of an invasion into the myometrium [10].

About 3% of endometrial cancer cases occur in women with an autosomal dominant hereditary predisposition to cancer, known as Lynch syndrome. This syndrome is the result of germline mutations in one of the mismatch repair proteins, MLH1, MSH2, MSH6, PMS1, and PMS2 [11].

To improve survival, it is essential to determine an adequate treatment strategy, which depends on the accuracy of the staging and, accordingly, risk stratification. In this regard, a reliable determination of the status of lymph nodes and invasion of the lymphovascular space appears to be necessary. To date, a sufficient number of diagnostic criteria for risk stratification in endometrial cancer have been developed; however, their specificity appears to be insufficiently accurate. Currently, medical–genetic factors for predicting and monitoring the course of malignant neoplasms are actively being developed and implemented. In this regard, studying contemporary scientific works in this field appears to be relevant.

The aim of this study was to analyze and systematize data from the scientific literature on the prognostic significance of medical–genetic factors and risk stratification predictors concerning endometrial cancer.

## 2. Materials and Methods

A literature review was conducted using data from 44 randomized clinical research studies, 7 systematic reviews, and 2 meta-analyses from the electronic databases of PubMed, Google Scholar, and Wiley Cochrane Library for the period from 2018 to 2023 using the keywords endometrial cancer, endometrial carcinoma, medical–genetic factors, DNA methylation, lymphadenectomy, microRNA expression, tumor marker, and genetic profiling.

## 3. The Role of Lymph Node Status and Lymphovascular Space Involvement in the Appropriateness of Performing Lymphadenectomy

Currently, almost all risk stratification systems for endometrial cancer are used, including the stage, tumor grade, histological type, and deep myometrial invasion [12].

The invasion of the lymphovascular space is an important initial stage of tumor metastasis and is defined as the invasion of tumor cells into lymphatic and/or blood vessels [13,14].

The study by Siegenthaler F et al. (2023) demonstrated that the presence of lymphovascular space invasion reduces disease-free survival among patients with MMRd, p53abn, and NSMP endometrial cancer, characterized by the absence of a specific molecular profile in the tumor, as well as the overall survival in patients with the molecular subtypes of p53abn and NSMP cancer. Invasion in NSMP cancer is an independent predictor of recurrent disease. The analysis of the obtained data also showed that the presence of invasion may be a significant predictor of recurrence in patients with MMRd endometrial cancer, which aligns with the ESGO/ESTRO/ESP risk classification. However, it is worth noting that the presence of lymphovascular space invasion is not associated with a worse prognosis in patients with stage IA p53abn and/or non-endometrioid endometrial cancer [15].

The assessment of the lymph node is critically important for choosing the treatment strategy for endometrial cancer [16,17]. Lymphovascular space invasion occurs in approximately 15% of patients at an early stage [18] and is reported to occur in around 25% of patients when all stages are included [19].

According to Benedetti Panici et al. (2008), pelvic lymphadenectomy has been shown to have better surgical staging, but systematic lymphadenectomy has not demonstrated improved disease-free or overall survival. In systematic pelvic lymphadenectomy, about 12–13% of patients with endometrial cancer have positive lymph nodes [20].

The role of lymphadenectomy was established in a 1987 GOG study, in which surgical intervention, including the dissection of pelvic and paraaortic lymph nodes, was performed on 33 patients with uterine body cancer. The frequency of lymph node involvement in this case correlated with the degree of tumor differentiation and the depth of invasion into the myometrium. This study served as the basis for the initial staging system for endometrial cancer in the International Federation of Gynecology and Obstetrics (FIGO) in 1988 [21].

Retrospective reviews indicate the therapeutic effect of lymphadenectomy in cases of metastatic involvement. In patients with poorly differentiated endometrial cancer, the removal of more than 11 pelvic lymph nodes was associated with improved overall survival compared to the removal of fewer than 11 pelvic lymph nodes [22]. A similar effect was also observed in patients at high risk of metastasis to the lymph nodes, including morphological types such as clear cell, papillary serous, and poorly differentiated endometrioid adenocarcinoma [23]. A large retrospective cohort analysis also demonstrated improved survival associated with pelvic and paraaortic lymphadenectomy in patients with intermediate and high-risk disease but not in patients with low-risk disease. Population data (SEER) also demonstrated a survival advantage associated with more extensive lymphadenectomy in high-risk endometrial cancer, but not in low-risk cases [24].

However, the performance of lymphadenectomy is controversial due to the risk of serious complications and the deterioration of quality of life for some patients.

Preoperative diagnosis of lymphovascular space invasion is challenging during preoperative examination and is detected postoperatively through histological analysis [25].

According to the recommendations of the National Comprehensive Cancer Network (NCCN), lymphadenectomy is recommended for adequate staging and should be performed to assess individual risk.

Therefore, a practical and accurate method for assessing metastasis to lymph nodes without invasive intervention is required, especially in cases of low recurrence risk.

## 4. Laboratory and Diagnostic Methods for Assessing Lymph Node Status

Currently existing methods for evaluating secondary lymph node involvement have insufficient diagnostic value.

Yes, the carbohydrate antigen 125 (CA-125) tumor marker is often elevated in the blood of patients with advanced stages of endometrial cancer and large tumor volumes, but it is not associated with metastasis to lymph nodes in the early stages [26]. Diagnostic imaging tests, such as computed tomography (CT), magnetic resonance imaging (MRI), and positron emission tomography (PET), are not sufficiently sensitive for detecting micrometastases [27,28]. Histological type, invasion into the myometrium, tumor diameter ≥ 2 cm, and extragenital diseases are risk factors for metastasis to lymph nodes; however, preoperative and intraoperative diagnostics using these criteria are imprecise and inadequate in clinical practice [29].

Procedures using sentinel lymph nodes are rapidly gaining popularity in the treatment of endometrial cancer. In a prospective cohort study (the study of fluorescent imaging for the robotic biopsy of the endometrial sentinel lymph node [FIRES]), the test characteristics of the sentinel lymph node biopsy were evaluated in patients with stage I disease according to FIGO of any histological subtype.

It was expected that the sentinel lymph node (SLN) mapping would be as accurate as it has been shown to be in breast cancer, but the pattern of lymphatic drainage in the uterus is more complex than that of the breast. Recent FIRES studies evaluated the sensitivity and negative predictive value of sentinel lymph node assessment compared to the gold standard of complete lymphadenectomy for detecting metastatic lymph node involvement in endometrial cancer. In this study, surgeons administered 0.5 mg of indocyanine green both superficially and deeply into the stroma of the cervix at the three o’clock and nine o’clock positions, totaling 1 mg. All the patients underwent hysterectomy with adnexa and pelvic lymphadenectomy, while paraaortic dissection was performed in 74% of cases at the surgeon’s discretion. The results of this study demonstrated a >99% negative predictive value of negative SLN biopsy and 3% missed lymph node dissections when SLN biopsy was performed without lymphadenectomy. At the same time, one false-negative result was identified in a patient with serous cancer, which carries a higher risk of metastasis to lymph nodes, including isolated paraaortic metastases. This raises the question of whether this approach is relevant for high-risk histological endometrial malignancies.

The current NCCN recommendations have approved SLN mapping as a method for endometrial cancer, with a level of evidence and consensus rated at the category 2B. SLN mapping may be considered for individual patients during the surgical stage of apparent uterine malignant neoplasm when metastasis has not been demonstrated through the imaging of extramural spread during the examination [30].

## 5. Medical–Genetic Factors of Metastatic Lymph Node Involvement and Lymphovascular Space Invasion

An alternative approach is to conduct a molecular analysis of primary cancer cells obtained from patients, as cancer cells in the primary tumor should be dissected regardless of LN+/LN− status [31].

Emiko Yoshida et al. (2017) conducted an examination of gene expression in endometrial cancer using a SAGE analysis, which quantitatively determined the level of expression promoters across the entire genome. Fourteen profiles delineated specific transcriptional networks between cases of positive and negative metastasis to lymph nodes. A sequential quantitative reverse transcription polymerase chain reaction (qRT-PCR) analyzed 115 primary tumors and showed that mRNA SEMA3D and the isoforms of TACC2, expressed through a new promoter, emerged as promising biomarkers with high accuracy when used in combination. The obtained data, taking into account all levels of recurrence risk, confirmed a significant association between mRNA SEMA3D and the new isoforms of mRNA TACC2 with LN +/LN− status in cases with a low-intermediate recurrence risk. At the same time, SEMA3D plays a role in inhibiting lymphangiogenesis, and a correlation has been found between high recurrence-free survival and the increased expression of this biomarker in an independent cohort (a RNA sequencing dataset created by TCGA). Based on the Kaplan–Meier analysis, a longer recurrence-free survival was found with the relative expression of SEMA3D compared to the isoform TACC2 (*p* = 0.048). The authors also compared the effectiveness of the studied biomarkers with other methods for diagnosing metastatic lymph node involvement, such as sentinel lymph node mapping and preoperative diagnostic tests based on imaging. Within the low and intermediate risk group, the preoperative imaging-based diagnostics (such as CT, MRI, and PET) correctly identified three out of eight positive cases of lymph node metastasis, whereas when using optimal values, the studied biomarkers accurately detected all eight cases. This result highlights the limitations of image-based diagnostics. In the course of the FIRES study, an evaluation of the effectiveness of sentinel lymph node mapping was conducted in a cohort of 385 cases, regardless of the current study, where a sensitivity of 97.2% and a negative predictive value of 99.6% were reported, which merits a comparison with the effectiveness of the biomarkers being studied. One of the clinically significant differences between the sentinel lymph node mapping and this approach is the degree of invasion: sentinel lymph node mapping requires a cervical injection of indocyanine green and the removal of the sentinel lymph node after the visualization (mapping). The FIRES study reported that 5.7% (22/385) of patients experienced serious adverse effects, with one of them being related to the investigational intervention.

Thus, high-resolution transcriptomics provides the confirmation of clear molecular profiles based on lymph node metastasis status for endometrial cancer, expanding opportunities for intraoperative diagnostics while reducing unnecessary surgeries in patients with minimal recurrence risk [32].

Watanabe T et al. (2019) aimed in their study to investigate candidate genes as potential diagnostic biomarkers and to determine whether they are predictors of lymphovascular space invasion in patients with uterine body cancer. To assess the hypothesis regarding the influence of gene expression profiles in primary endometrial carcinoma on the determination of the presence or absence of lymphovascular space invasion, the authors conducted a DNA microarray analysis using 88 tumor samples obtained from patients with endometrial carcinoma who underwent surgical treatment at Fukushima Medical University Hospital between 2010 and 2015. Fifty-five candidate genes were identified that were significantly differentially expressed between 26 positive and 62 negative samples with lymphovascular space invasion, according to Student’s *t*-test (*p* < 0.005). The functional category of these genes included genes related to the cell cycle (n = 20) and genes related to DNA repair (n = 8). Taking into account hierarchical clustering, the accuracy of the signature of 55 genes was assessed: sensitivity, specificity, positive predictive value, negative predictive value, and accuracy were 92%, 63%, 51%, 95%, and 72%, respectively. The data from this study showed that the signature of 55 genes may contribute to predicting lymphovascular space invasion in endometrial cancer and provide clinically important information for better treatment [25].

Shinichi Togami et al. (2019) published preliminary data from a quantitative analysis of real-time PCR for detecting metastases in lymph nodes in endometrial cancer in their study. The authors investigated the tissue of primary tumors, cancerous tissues, and resected pelvic lymph nodes of 105 patients with endometrial cancer through a real-time PCR analysis to determine the copy number of CK19 mRNA in endometrial cancer tissues, as well as in negative and positive metastatic lymph node samples. Next, the PCR results were compared with the data from the pathological examination. The expression of mRNA CK19 was found in 98% (104/106) of tumors, with an average copy number of 3.0 × 10^5^/μL. Twelve LNs were identified as positive in the pathological test. The expression of mRNA CK19 was higher in metastatically affected lymph nodes than in lymph nodes without metastatic involvement (*p* < 0.01); the pathological data and PCR results showed no discrepancies. When the threshold value was set at 4500 copies/mL, the real-time PCR analysis using CK19 mRNA demonstrated high sensitivity and specificity [33].

Another group of researchers demonstrated that the HOXB9 gene, a transcription factor of the HOX family, is overexpressed in the tissues of primary endometrial cancer tumors, contributing to the progression of endometrial cancer by affecting the oncogenic protein E2F3, the expression of which correlates with metastatic lymph node involvement. Junhu Wan et al. (2018) analyzed the expression of HOXB9 in a series of 88 samples of endometrial carcinoma tumor tissue, 15 normal samples of endometrium in the proliferative phase, and 21 samples of atypical endometrial hyperplasia using immunohistochemistry. The authors found that the expression level of HOXB9 in normal proliferative endometrium, atypical endometrial hyperplasia, and endometrial carcinoma gradually increased (*p* = 0.0196). In endometrial carcinoma, the expression of HOXB9 correlated only with the histological grade (*p* = 0.0081) and the status of metastasis in lymph nodes (*p* = 0.001). The expression level of HOXB9 in G1, G2, and G3 gradually increased, and the tumors with metastases expressed significantly higher levels than the tumors without metastases. Additionally, a high level of HOXB9 expression was reliably correlated with shorter disease-related survival times. Furthermore, the results from the TCGA database, which were primarily analyzed using web-based databases, interactive gene expression profiling analysis (“GEPIA, (accessed on 12 April 2017)” http://gepia.cancer-pku.cn/) and the “LinkedOmics database (accessed on 7 November 2017) (http://www.linkedomics.org), showed that the HOXB9 expression was significantly elevated in endometrial cancer tissues compared to normal tissues, and the increased level of HOXB9 expression corresponds to the decrease in the overall survival in endometrial cancer. The obtained data indicate that HOXB9 is highly expressed, and elevated levels of HOXB9 may predict metastatic lymph node involvement and poor outcomes for patients [34].

Sukbum Kang et al. (2018) developed a predictive model using the expression signature of 12 genes to identify patients with a low risk of lymph node metastasis in endometrioid-type uterine cancer. Datasets from 330 patients with histologically confirmed endometrioid carcinoma of the endometrium from the Total Cancer Care (TCC) consortium network data in Florida hospitals (including the H. Lee Moffitt Cancer Center) and eight other national centers, who underwent lymphadenectomy during the surgical staging procedure, were divided into three sets in chronological order. Totals of 110, 112, and 108 patients were divided into the preparatory set, the evaluation set, and the validation set, respectively. The average age of the patients was 63 years (ranging from 29 to 90 years). The average number of lymph nodes extracted was 28 (ranging from 1 to 38), and paraaortic lymphadenectomy was performed in 118 out of 330 patients (35.8%). Metastases in lymph nodes were found in 45 out of 330 patients (13.6%). Using datasets for training and evaluation, the authors developed the signature of 12 genes that allows for the prediction of metastasis to lymph nodes. These were the genes GREM2, FMO2, TMEM212, ESR1, RPTN, PRR9, TCHHL1, CPB1, CLCN2, ITLN2, PKHD1L1, and SLC9C2. In the multivariate logistic regression analysis, the researchers found that the linear model estimates of the signature from 12 genes demonstrated a sensitivity of 100% and a specificity of 42%. Thus, a 12-gene signature can be useful for identifying patients with a very low risk of lymph node metastasis in endometrial cancer. This model can help patients with high clinical risk factors avoid unnecessary lymphadenectomy [35].

A similar study for thyroid carcinoma to identify the relationship between gene expression and metastasis to regional lymph nodes was conducted by a group of researchers led by Hammad MO et al. (2019). The authors studied the clinical significance of the genes CEP78 and WDR62 in differentiated thyroid carcinoma with a high risk of metastasis to lateral cervical lymph nodes. Using a quantitative real-time polymerase chain reaction, the expression of mRNA CEP78 and WDR62 was assessed in 40 samples of thyroid carcinoma tissue and 40 samples of goiter tissue. These results were compared with the clinical, ultrasound, laboratory, and pathological data of the patients to analyze the relationship between these characteristics and metastasis to lateral cervical lymph nodes. The results showed that the relative levels of CEP78 mRNA were significantly lower in thyroid cancer tissues than in goiter tissues (*p* = 0.002). In the binomial (multifactorial) logistic regression analysis, a significant factor for predicting metastases in lateral cervical lymph nodes was the low expression of mRNA CEP78 (cut-off value ≤ 0.54; *p* = 0.03). CEP78 may serve as a promising molecular biomarker for differentiating between thyroid carcinoma and goiter tissues, and, furthermore, it could act as a predictor of metastasis to lateral cervical lymph nodes [36].

According to the study by van den Heerik et al. (2021), TCGA identified four molecular subclasses of endometrial cancer based on somatic mutation burden and the nature of copy number alterations. Thus, ultra-mutated cancer with mutations in the exonuclease domain of DNA polymerase epsilon (POLE), hypermutated endometrial cancer with microsatellite instability, endometrial cancer with a high copy number and frequent TP53 mutations, and a group of endometrial cancers with low copy numbers were distinguished. The definition of the molecular type of cancer has prognostic significance.

Thus, POLE mutations are typically found in cases of high malignancy with lymphocytic infiltration; however, the prognosis for such patients is favorable, with a low likelihood of recurrent disease, regardless of the adjuvant therapy administered. Thus, tumor neoantigens caused by ultramutation provoke a pronounced cytolytic immune response that impairs the functioning of cancer cells and leads to a reduction in metastatic potential.

Microsatellite instability is associated with a deficiency in the nuclear expression of one or more repair factors, leading to the accumulation of mismatches, insertions, and deletions. Endometrial cancer with repair deficiency triggers a pronounced immune response and is characterized by an intermediate prognosis.

The third group includes tumors characterized by high variability in the number of somatic copies and a relatively low occurrence of somatic mutations but with frequent TP53 mutations. This group mainly includes non-endometrioid types of cancer with a poor prognosis due to aggressive growth, early metastasis to the lymphovascular space, and rapid disease progression (serous carcinoma, carcinosarcoma, about half of clear cell carcinoma cases, and poorly differentiated endometrioid adenocarcinomas).

The last of the aforementioned molecular subgroups of endometrial cancer is characterized by the highest prevalence. This group has a high heterogeneity. One of the promising prognostic unfavorable markers is the presence of mutations in exon 3 of β-catenin (CTNNB1) [37].

**Figure 2:**
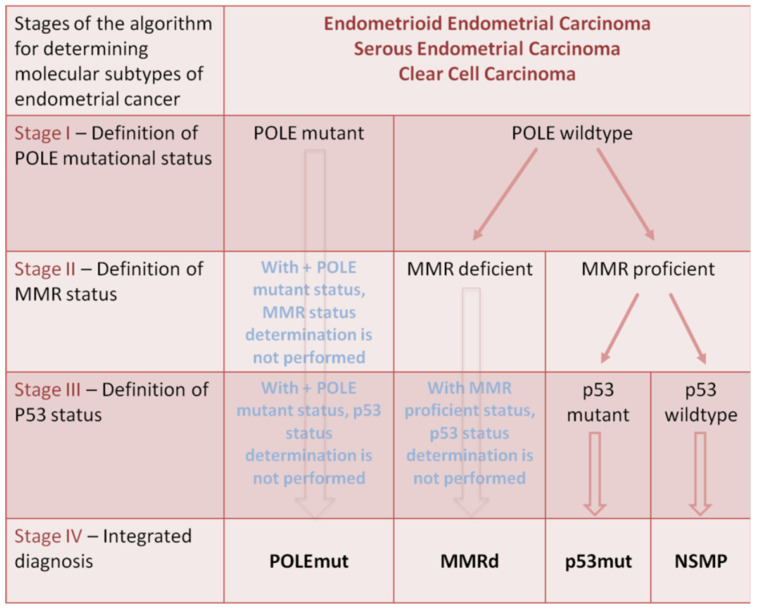
Stages of molecular tumor subtypes diagnostic in patients with histologically confirmed endometrioid, serous, and clear cell endometrial cancer.

Figure 2 presents the diagnostic algorithm for the four molecular subtypes of endometrial cancer. In the first stage, in patients with histologically confirmed endometrioid, serous, and clear cell endometrial cancer, the presence of POLE mutations in cancer cells is determined. In the case of a positive result, the molecular subtype of the tumor is classified as POLEmut. In the absence of a POLE mutation, the MMR status of the tumor cells is determined. In the case of a deficiency in the repair system of unpaired DNA, the molecular subtype of the tumor corresponds to MMRd, and no further investigation is conducted. In the absence of such a deficiency, the presence of a p53 mutation is determined at the third stage, and the molecular subtype is classified as p53mut if it is present, or as NSMP.

In the study by Wang T et al. (2023), it was shown that with an increased expression of CD8, CD4, PD-L1, or Foxp3 in POLE mutant endometrial cancer, survival time was significantly prolonged compared to the subgroup with downregulated or wild-type POLE. In the future, regulating lymphocytic infiltration and inhibiting genes such as PD-L1 may serve as a new treatment strategy for this type of tumor [38].

Alicia León-Castillo et al. (2020) studied the advantages of combined adjuvant chemoradiotherapy compared to radiotherapy in a randomized trial involving women with high-risk endometrial cancer. (PORTEC-3). The authors used the molecular classification of endometrial cancer presented in the Cancer Genome Atlas as the most clearly prognostically valuable. Immunohistochemical studies for p53 proteins and the mismatch repair (MMR) system, as well as DNA sequencing for the exonuclease domain of POLE, were conducted on biopsy material from 423 patients. Based on this, the subtypes of endometrioid cancer were classified as abnormal p53 (p53abn), POLE ultra-mutated endonuclease (POLEmut), mismatch repair deficient (MMRd), or non-specific molecular profiles (NSMP). The five-year recurrence-free survival rates for the subgroups of endometrial cancer were as follows: 48% for p53abn, 98% for POLEmut, 72% for MMRd, and 74% for NSMP (*p* < 0.001). The five-year disease-free survival after the combined adjuvant chemotherapy and radiation therapy compared to the radiation therapy alone for the subtype of endometrioid cancer were as follows: 59% versus 36% for p53abn (*p* = 0.019); 100% versus 97% for patients with POLEmut (*p* = 0.637); 68% versus 76% (*p* = 0.428) for MMRd; and 80% versus 68% (*p* = 0.243) for NSMP. Molecular classification has demonstrated a high prognostic value in high-risk endometrial cancer, with significantly improved recurrence-free survival with adjuvant chemoradiotherapy for tumors with abnormal p53 (p53abn), regardless of the histological type [39].

**Figure 3:**
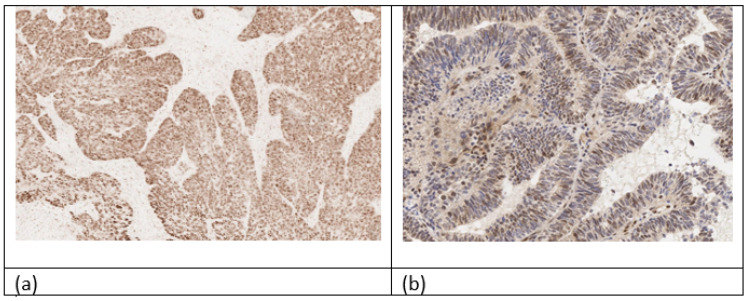
Immunohistochemical study with p53 (**a**)—hyperexpression of p53 in serous carcinoma (p53 mutant), (**b**)—25% expression of p53 in endometrioid carcinoma low grade G2 according to FIGO (p53 wildtype).

In conducting an immunohistochemical study with abnormal p53, the following morphological features were observed: 80% strong and diffuse nuclear staining, complete absence of nuclear staining in all cells, and moderate or strong cytoplasmic staining. On the other hand, in the wildtype, the diffuse nuclear staining of p53 was observed [40]. Figure 3 shows the digitized immunohistochemical studies of the histological samples from the authors’ personal archive. Figure 3a—p53 (wildtype, 25%), low-grade G2 endometrioid carcinoma; Figure 3b—hyperexpression of p53 in serous carcinoma.

Riedinger CJ et al. (2023), in their study, first demonstrated that the epigenetic defect in MMR is a predictor of metastasis and recurrences in lymph nodes, regardless of the tumor volume [41].

**Figure 4:**
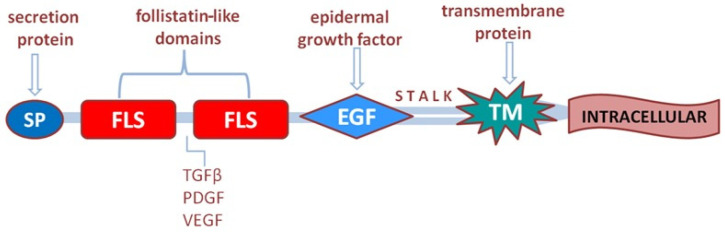
TMEFF2 gene structure and its relationship with follistatin and epidermal growth factor domains.

In the study by Horie M et al. (2000), the relationship of the TMEFF2 gene—a transmembrane protein—with the epidermal growth factor (EGF) and two follistatin-like domains, hyperplastic polyp 1 (HPP1), or the transmembrane protein TENB2 was analyzed [42]. The structural domains of follistatin in the extracellular domain of TMEFF2 bind and regulate numerous growth factors, including the TGFβ family, PDGF, and VEGF [43] (Figure 4).

Several studies have been conducted on TMEFF2 in the oncological diseases of the female reproductive system, focusing on the methylation of TMEFF2 in endometrial cancer, high-grade squamous intraepithelial lesions (HSIL), and cervical cancer [44,45].

Lingling Gao et al. (2020) used TCGA to study the expression of TMEFF2 in various types of endometrial cancer, which included a total of 354 endometrial cancer samples, 25 normal endometrial samples, and 338 blood samples. The results showed that the number of copies of TMEFF2 DNA in serous endometrial carcinoma and mixed endometrial carcinoma was significantly higher than in the control group (*p* < 0.05). Additionally, the levels of the methylation of the TMEFF2 gene were analyzed, and it was found that, regardless of the type of sample, age, race, weight, cancer stage, and tumor differentiation grade, the TMEFF2 exhibited low levels of methylation; however, these levels were higher than those in healthy individuals. A total of 75 cases of endometrial cancer were divided into groups with low (-/+) and high (++/+++) expressions of TMEFF2 based on the TMEFF2 expression in endometrial cancer tissues. The results revealed a high level of positive expressions of TMEFF2 in patients with stage III-IV endometrial carcinoma, at 95% (18/19), which was higher than in patients with stages I-II (66%, 37/56) (*p* < 0.05). The high expression rate of TMEFF2 gradually increased as the degree of differentiation decreased. The high expression rate in the poorly differentiated group was 87.50% (29/32); in the well-differentiated group, it was 47.37% (9/19), and in the moderately well-differentiated group, it was 62.79% (27/43) (*p* < 0.05). TMEFF2 was highly expressed in endometrial cancer tissues, and its high expression was associated with the FIGO stage, degree of differentiation, and lymph node metastasis. In the group of metastatic lymph nodes, the high level of TMEFF2 expression was 100% (14/14), which was higher than in the group without metastases (68.75%, 33/48) (*p* < 0.05). The univariate Kaplan–Meier analysis showed that the overall recurrence-free survival was significantly lower in patients with endometrial cancer with a high TMEFF2 expression compared to patients with a low TMEFF2 expression (*p* = 0.015).

The expression of TMEFF2 was also determined in three lines of endometrial cancer cells (Ishikawa, HEC-1A, and HEC-1B). The results showed that the expression of TMEFF2 in the Ishikawa cell line was higher than in the other two cell lines, and the expression of TMEFF2 in the Ishikawa cells was inhibited by RNA interference. The ability to proliferate decreased after the inhibition of TMEFF2 expression in the Ishikawa cells (*p* < 0.05). The data from this research indicate that the expression of TMEFF2 is associated with the development and progression, as well as the prognosis, of endometrial cancer, and may also assist in identifying therapeutic targets for the endometrium [46].

A group of researchers led by Juan Wang et al. (2018) assessed the expression of delta-like protein 3 (DLL3) as a tumor marker and prognostic predictor for endometrial cancer [47]. DLL3 is an atypical member of the Notch receptor ligand family that can inhibit the activation of Notch receptors [48]. Based on the TCGA data, information was obtained from 545 patients with primary endometrial cancer, and tissue samples from these patients were profiled for differentially expressed genes. Histologically, the majority of the tumors were endometrioid adenocarcinomas (74.9%, n = 408), followed by serous adenocarcinomas of the endometrium (21.1%, n = 115), while the remaining tumors were classified as “mixed” (4%, n = 22). Patients with stage I, II, III, and IV cancer accounted for 62.62%, 9.58%, 22.84%, and 4.96% of the cohort population, respectively, while patients with tumors of grades 1, 2, and 3 differentiation represented 18.5%, 22.7%, and 58.8%, respectively. Almost half of all the patients had a deep myometrial invasion (49.6%), and 10% and 5% of these patients had tumor metastases in pelvic and paraaortic lymph nodes, respectively. The average observation period was 30.3 months. The level of DLL3 expression was significantly increased in the endometrial cancer tissue compared to the non-tumor tissue (*p* < 0.0003). The authors found that the increased regulation of DLL3 expression was associated with an older patient age (≥64 years), a higher stage of tumor according to the International Federation of Gynecology and Obstetrics (FIGO) (OR = 2.9 for stage I/II compared to stage III/IV), a lower differentiation grade 3 (OR = 5.1 for grade 1/2 compared to grade 3), a deep invasion into the myometrium (OR = 2.2), and metastases to pelvic (OR = 12.9) and paraaortic lymph nodes (OR = 9.9) (*p* ≤ 0.001). Kaplan–Meier curves and the log-rank test showed that the increased expression of DLL3 is also associated with shorter overall survival in patients with endometrial cancer (*p* = 0.0045). Current data have demonstrated that DLL3 expression may be a potential new oncological marker for early diagnosis and prognosis in patients with endometrial cancer [47].

Wei Bao et al. (2019) assessed the expression of the microRNA gene 107 (miR-107-5p) using real-time PCR in 71 patients with endometrial cancer and found that miR-107-5p was significantly elevated in the tumor tissues of endometrial cancer compared to the normal endometrium. The increased regulation of miR-107 was associated with a higher FIGO stage (*p* < 0.05), less pronounced histological differentiation (*p* < 0.05), invasion into the myometrium (*p* < 0.001), and metastasis to lymph nodes (*p* < 0.01). The data from the study showed that miR-107 promotes the proliferation and invasion of endometrial cancer cells. Then, the authors discovered that miR-107 can directly bind to the 3′ untranslated region of the mRNA of the estrogen receptor in endometrioid carcinoma cells and inhibit the expression of α-estrogen receptors at the mRNA and protein levels in the cells. Thus, miR-107 plays an important role in the development of endometrial carcinoma through estrogen α-receptors [49].

The work of Ahmed EA et al. (2022) shows that miR-202 may serve as a prognostic biomarker for various types of cancer. Thus, the increase in miR-202 regulation correlates with drug resistance in breast cancer, acts as a tumor suppressor in the gastrointestinal tract, and plays a significant role in colorectal cancer and non-small cell lung cancer. The decrease in miR-202 levels in the tumor tissues of patients with endometrial cancer is a poor prognostic factor, which is associated with the suppressive role of miR-202 in this type of cancer [50].

Kong J et al. (2019), in their study, found that miR-29b inhibits the proliferation and reduces the migration and invasion of endometrial cancer cells. The proliferation of endometrial cancer cells was assessed using a water-soluble tetrazolium (WST)-1 analysis, while transwell migration and invasion assays with Matrigel were used to evaluate the effect of miR-29b on the migration and invasion of cancer cells. Thus, it has been found that, through the direct regulation of PTEN (binding to the 3′-untranslated region of PTEN), miR-29b has a strong impact on cell migration and invasion. The study also revealed that the expression of miR-29b is associated with the increased sensitivity of endometrial cancer cells to cisplatin and the cisplatin-induced enhancement of apoptosis through the regulation of BAX and Bcl-2 expression [51].

Sun X et al. (2021) assessed the expression of miR-501 in patients with endometrial cancer. The expression of miR-501 was determined using the real-time PCR method. The proliferative capacity was assessed using a MTT analysis, colony formation analysis, and cell cycle analysis. A transwell analysis was also used to assess the migration and invasion of cancer cells. Using a luciferase analysis, quantitative real-time PCR, and western blotting, it was determined that HOXD10 is a target gene of miR-501. Using the methods mentioned above, researchers were able to identify that the high expression of miR-501 is associated with a higher risk of metastasis to pelvic lymph nodes and shorter overall survival in high-grade endometrial cancer [52].

In the studies by Xiong H et al. (2021), it was demonstrated that miR-199a/b-5p is associated with the inhibition of the migration and invasion of endometrial cancer cells by suppressing factors related to EMT and the EMT signaling pathway. It was also found that the FAM83B protein may promote metastasis by weakening the inhibition of the cell migration and invasion induced by miR-199a/b-5p. Thus, scientists suggest evaluating the expression of the aforementioned factors collectively for a more accurate assessment of risk stratification [53].

Wang J et al. (2019) assessed the expression of miR-135a in endometrial cancer and analyzed its impact on the proliferation, chemotherapy sensitivity, migration, and invasion of endometrial cancer cells. The study demonstrated a connection between miR-135a and the increased proliferation and invasion of the tumor process, as well as showing that this marker inhibits the apoptosis induced by cisplatin in endometrial cancer cells by regulating the expression of BAX and Bcl-2 [54].

Jia Bian et al. (2020) conducted a study to identify new genes associated with tumor grade with potential implications for the prognosis and progression of uterine body cancer. A total of three datasets of gene expression microchips were downloaded from the Gene Expression Omnibus database, and one RNA sequencing dataset with corresponding clinical information about patients with endometrial cancer was obtained from the Cancer Genome Atlas database. Thus, 1447 differentially expressed genes were identified between malignant endometrial tissues and normal endometrial tissues. The analysis of the weighted co-expression gene network was conducted to assess the relationship between differentially expressed genes and clinical features. Five genes closely associated with oncogenesis and the prognosis of the endometrioid carcinoma of the uterine body have been identified. Among them, serine/threonine kinase B mitotic checkpoint (BUB1B), cyclin B1 (CCNB1), cell division cycle protein 20 (CDC20), and non-SMC condensin I complex subunit G (NCAPG) were involved in the pathways regulating the cell cycle, while DLG-associated protein 5 (DLGAP5) was involved in the Notch receptor. These genes are highly expressed in cancerous endometrial tissues compared to normal endometrial tissues at the protein level. Moreover, the higher expression of these genes predicted a greater degree of tumor malignancy, a higher risk of metastasis, and worse overall survival. Thus, the study identified five gene signatures that can be used to predict progression [55].

Liu T et al. (2023) analyzed the expression of KIF18A and its role as a prognostic marker in various types of cancer. It has been found that the mutation frequency of this gene is the highest in endometrial cancer. The study showed that KIF18A positively correlates with the Ki67 proliferation index, indicating that KIF18A may be associated with the acceleration of tumor cell division. A positive correlation has also been established between KIF18A expression and Th2 for 33 types of cancer in the TCGA database (Th2 suppresses the anti-tumor immune response). It is presumed that KIF18A interacts with tumor and immune cells, as indicated by its positive correlation with several genes such as PD-L1, PD-1, CTLA4, TIGIT, HAVCR2, PDCD1LG2, and LAG3. Researchers found that the expression of KIF18A was positively associated with regulatory genes of RNA methylation in certain types of cancer. These data showed that KIF18A may contribute to oncogenesis through RNA methylation [56]. Weiwei Luo et al. (2018) suggested that KIF18A is associated with the invasion and metastasis of cancer cells through a pathway related to MMP-7/MMP-9 [57]. In turn, the study by Weiwei Chen et al. (2021) associates the expression of proteins from certain subtypes of MMP with cervical cancer [58]. Thus, KIF18A may potentially become a new prognostic marker for many types of cancer, including endometrial cancer.

Feng X et al. (2023) examine the possibility of applying a new nomogram model for risk stratification and metastasis prediction in endometrial cancer. This nomogram considers not only the commonly accepted criteria (tumor invasion depth, lymphovascular infiltration, histological type, grade, etc.) but also includes the assessment of metabolic factors (BMI, PP, FBG, TG, and HDL). Compared to the Mayo criteria, the use of this method is characterized by greater accuracy and specificity [59].

Table 1 shows data from studies with correlations between the expression of the main biomarkers considered and the results in accordance with changes in their expression.

## 6. Conclusions

Molecular genetic profiling methods have been increasingly used in recent years for stratifying the risk of the progression of endometrioid carcinoma. Within the framework of such an approach, gene expression signatures are used as tools for predicting the course and outcomes of malignant neoplasms and particularly considered as diagnostic markers for the risk of tumor invasion into the lymphovascular space and metastasis to lymph nodes. The application of these methods is particularly relevant for patients with a low risk of lymph node metastases in endometrioid endometrial cancer, as their prognostic characteristics, especially the high negative predictive value, allow for the avoidance of systematic lymphadenectomy, which can lead to serious complications and a deterioration in the quality of life for patients.

Further study and the implementation of molecular–biological methods for pre- and intraoperative diagnosis related to risk stratification in endometrial cancer, including the assessment of the prognostic significance of endometrial cancer metastasis to regional lymph nodes, is necessary for determining the potential prognosis and progression of the disease. The results of using such an approach play a key role in choosing the treatment strategy: intraperative surgical treatment and adjuvant therapy with the aim of minimizing the postoperative negative consequences of lymphadenectomy in cases with a low risk of recurrence, as well as reducing the duration of primary surgical treatment for patients. The application of these methods also allows for accelerated postoperative rehabilitation and contributes to a faster initiation of subsequent adjuvant therapy, increasing the effectiveness of combined treatment and the overall survival of patients with endometrial cancer.

## Figures and Tables

**Table 1:** The main biomarkers of the studies, their expression and the result of its changes.

Study	Biomarker	Expression	Results of Biomarker Expression Changes
Emiko Yoshida et al. (2017) [30]	mRNA SEMA3D	↑	inhibition of lymphangiogenesis, increased relapse-free survival
	relative expression of SEMA3D compared to TACC2 isoform	↑↑	longer relapse-free survival elapse
Shinichi Togami et al. (2018) [31]	Cytokeratin 19 mRNA (CK19)	↑	metastatic lymph nodes marker
Junhu Wan et al. (2018) [32]	HOXB9	↑	higher risk of progression, metastasis, and shorter survival time
Sukbum Kang et al. (2019) [33]	GREM2, FMO2, TMEM212, ESR1, RPTN, PRR9, TCHHL1, CPB1, CLCN2, ITLN2, PKHD1L1 и SLC9C2	↑	low risk of lymph node metastasis
van den Heerik et al. (2023) [35]	POLEmut	↑	decreased metastatic potential, low probability of recurrence
	MMRd	↑	pronounced immune response with an intermediate prognosis
	p53abn	↑	non-endometrioid types and endometrioid adenocarcinoma G 3 (serous cancer, carcinosarcoma, about half of clear cell cancer cases), early metastasis, unfavorable prognosis
Wang T et al. (2020) [36]	CD8, CD4, PD-L1 or Foxp3 mutants of POLE	↑	increased overall survival
Riedinger CJ et al. (2000) [38]	epigenetic defect of MMR	↑	predictor of metastasis and relapse to lymph nodes, regardless of tumor volume
Horie M et al. (2000) [39,40,41,42] Lingling Gao et al. (2018) [43]	TMEFF2	↑	increased risk of progression, metastasis to lymph nodes, decreased overall relapse-free survival
Juan Wang et al. (2011) [44,45]	DDL3	↑	deep myometrial invasion, metastasis to pelvic and paraaortic lymph nodes, higher tumor stage, lower grade of differentiation and shorter overall survival
Wei Bao et al. (2019) [46]	gene microRNA-107 (miR-107-5p)	↑	decreased histological differentiation, increased myometrial invasion and lymph node metastasis
Ahmed EA et al. (2022) [47]	miR-202	↓	poor prognostic factor
Kong J et al. (2019) [48]	miR-29b	↑	inhibition of proliferation and invasion, increased sensitivity to chemotherapy
Sun X et al. (2021) [49]	miR-501	↑	high risk of pelvic lymph node metastasis and shorter overall survival
Xiong H et al. (2021) [50]	miR-199a/b-5p	↑	inhibition of tumor cell migration and invasion
Wang J et al. (2019) [51]	miR-135a	↑	increased tumor invasion and proliferation
Jia Bian et al. (2020) [52]	BUB1B CCNB1 CDC20 NCAPG DLGAP5	↑	higher tumor grade, higher risk of metastasis, and worse overall survival
Liu T et al. (2023) [53] Weiwei Luo et al. (2018) [54] Weiwei Chen et al. (2021) [55]	KIF18A	↑	accelerated division of tumor cells with invasion and metastasis

## Data Availability

The data presented in this study are available upon request from the corresponding author.
